# Elevated gamma‐glutamyl transferase levels are associated with stroke recurrence after acute ischemic stroke or transient ischemic attack

**DOI:** 10.1111/cns.13909

**Published:** 2022-07-04

**Authors:** Siqi Li, Anxin Wang, Xue Tian, Yingting Zuo, Xia Meng, Yumei Zhang

**Affiliations:** ^1^ Department of Neurology, Beijing Tiantan Hospital Capital Medical University Beijing China; ^2^ China National Clinical Research Center for Neurological Diseases Capital Medical University Beijing China; ^3^ Department of Rehabilitation medicine, Beijing Tiantan Hospital Capital Medical University Beijing China

**Keywords:** gamma‐glutamyl transferase, ischemia, recurrence, stroke

## Abstract

**Aims:**

Gamma‐glutamyl transferase (GGT) is considered a marker of oxidative stress in vivo. In this study, we aimed to examine the association of serum GGT levels with 3‐month and 1‐year stroke recurrence in patients with acute ischemic stroke or transient ischemic attack (TIA).

**Methods:**

We conducted a large and multicenter cohort study. Participants with ischemic stroke or TIA who had a baseline GGT measurement were enrolled in the China National Stroke Registry‐3 study from August 2015 to March 2018. They were divided into four groups according to sex‐specific quartiles of GGT levels. The effect of GGT on stroke recurrence and other vascular events was examined during the 1‐year follow‐up period. Multivariate Cox regression models were performed to evaluate the association. Discrimination tests were used to examine the degree to which incorporating GGT into the conventional model predicted stroke adverse outcomes.

**Results:**

A total of 12,504 patients were enrolled. At both the 3‐month and 1‐year follow‐ups, patients in the highest quartile group of GGT levels exhibited a higher risk of stroke recurrence [HR 1.32 (95% CI 1.07–1.63), HR 1.34 (95% CI 1.13–1.60)], ischemic stroke [HR 1.37 (95% CI 1.10–1.71), HR 1.37 (95% CI 1.14–1.64)], and combined vascular events [HR 1.34 (95% CI 1.09–1.65), HR 1.34 (95% CI 1.13–1.59)] than those in the lowest quartile group. Moreover, the Kaplan–Meier curves revealed that the incidence rates of stroke adverse outcomes were quite different in the four groups. The highest quartile group showed the highest cumulative incidence, while the lowest quartile group showed the lowest cumulative incidence. After applying discrimination tests, adding GGT into the conventional model resulted in slight improvements in predicting stroke adverse outcomes (NRI: 10%–14%).

**Conclusion:**

This study demonstrated that elevated GGT levels were positively associated with an increased risk of stroke adverse outcomes, namely, recurrence, ischemic stroke, and combined vascular events.

## Graphical abstract

This large, multicenter cohort study reveals elevated GGT levels are positively associated with the increased risk of stroke adverse outcomes, namely, recurrence, ischemic stroke, and combined vascular events. There is a slight improvement in predicting the risk of stroke adverse outcomes when adding GGT into the conventional model after applying discrimination tests.

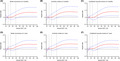

## 1 INTRODUCTION

Stroke is the leading cause of death and disability worldwide.^1^, ^2^, ^3^ Previous studies have shown that nearly 20% of stroke patients will have recurrence of stroke. Approximately 50% of recurrent strokes occur within a few days to a few weeks after ischemic cerebrovascular disease. In addition, recurrent stroke may lead to the deterioration of functional outcomes, decreased quality of life, and even increased mortality.^4^, ^5^, ^6^ Therefore, it is necessary to predict the risk of recurrence and new vascular events in stroke patients in a timely manner.

Gamma‐glutamyl transferase (GGT) is a recognized serum marker of alcoholic liver disease.^7^, ^8^, ^9^ It has been reported to be associated with the identification of a variety of diseases.^10^, ^11^ Multiple previous studies reported an association between GGT activity and cardiovascular disease events (CVDs) in healthy populations.^12^, ^13^, ^14^, ^15^, ^16^, ^17^, ^18^ Researchers have observed that people with higher GGT levels were more likely to develop stroke after a period of follow‐up than those with lower GGT levels. In addition, some studies have further focused on stroke patients to investigate the relationship between GGT levels and stroke. A study showed that GGT activity was more strongly associated with cardioembolic stroke among ischemic stroke patients, which was largely due to the presence of atrial fibrillation.^19^ Moreover, researchers further explored the association between GGT levels and adverse clinical outcomes of CVDs in stroke patients.^20^, ^21^, ^22^ Results showed that elevated serum GGT levels were associated with all‐cause or cardiovascular disease mortality after stroke and might be independent of alcohol consumption. However, the relationship between GGT activity and other stroke adverse outcomes, namely, recurrence, ischemic stroke, and combined vascular events, remains to be further elucidated. Interestingly, a previous study found that the significant association between GGT activity and stroke was no longer significant after adjustment for multiple factors, which meant that GGT activity might be less likely to predict the severity of cardio‐cerebrovascular diseases.^23^ Thus, the objectives of this study were to examine the association of serum biomarker GGT levels with stroke adverse outcomes, namely, recurrence, ischemic stroke, and combined vascular events, and to evaluate whether GGT levels can predict the risk of stroke adverse outcomes at the 3‐month and 1‐year follow‐ups.

## 2 METHODS

### 2.1 Study population

The China National Stroke Registry‐3 (CNSR‐3) study is a large multicenter study that includes approximately 201 hospitals and national registries in China and recruited consecutive patients from August 2015 to March 2018.^24^ We identified 15,166 participants who were selected from the CNSR‐3 study. The inclusion criteria for participants in this study were as follows: (i) patients diagnosed with acute ischemic stroke (AIS) or transient ischemic attack (TIA); (ii) patients hospitalized within 7 days from symptom onset; and (iii) patients who were older than 18 years. Among these patients, the following were further excluded: 2291 patients who lacked serum GGT data at baseline and 371 patients who were lost to follow‐up. Ultimately, the total number of eligible participants in the present study was 12,504.

The present study was performed in accordance with the guidelines described by the Helsinki Declaration and was approved by the Ethics Committees of Beijing Tiantan Hospital (No. KY2015‐001‐01). All participants signed a written informed consent form prior to their inclusion in the study.

### 2.2 Data collection

The baseline data of the enrolled participants were collected following the standard data collection protocol developed by the steering committee. The protocol and the statistical analysis plan have been published in a previous study.^25^

The information from all participants was collected by comprehensive assessments on admission and detailed medical records, including baseline characteristics (age, sex, body mass index, alcohol consumption, smoking status, etc.), medical histories (stroke or TIA, hypertension, cardiovascular disease, dyslipidemia, diabetes mellitus, peripheral vascular disease) and physical examination results (blood pressure, the modified Rankin Scale (mRS) score, the National Institutes of Health Stroke Scale (NIHSS) score). All imaging data were collected in DICOM format on disks and analyzed by two professional neurologists. Moreover, exposure to various medications during hospitalization (antiplatelet aggregation therapy, anticoagulation treatment, antihypertensive treatment) was assessed. In addition, serum GGT, fasting blood glucose (FBG), total cholesterol (TC), triglyceride (TG), alanine aminotransferase (ALT), and aspartate aminotransferase (AST) levels from fasting blood samples were collected carefully and with high quality within 24 h of admission to address potential sources of bias. All blood samples were stored in EDTA anticoagulant collection tubes and serum separation tubes in a −80°C refrigerator.

### 2.3 Outcome evaluation

The primary clinical outcome was stroke recurrence (defined as a new focal neurological impairment due to acute stroke events, including new ischemic stroke and hemorrhagic stroke, which was confirmed by neuroimaging). The secondary clinical outcomes were ischemic stroke and combined vascular events (nonfatal stroke, nonfatal myocardial infarction, cardiovascular death). The outcomes mentioned above were observed at 3 months and 1 year after stroke onset. According to the World Health Organization criteria, the diagnostic criteria for stroke rely on symptoms, physical signs, scale evaluations, and neuroimaging (magnetic resonance or brain computed tomography).^25^, ^26^

### 2.4 Statistical analysis

The data were tested for normal distribution using the Kolmogorov–Smirnov test. Continuous variables conforming to a normal distribution are presented as the means ± standard deviations (SDs) and were compared using ANOVA. Continuous variables that did not exhibit a normal distribution are presented as medians with interquartile ranges (IQRs) and were compared using the Kruskal–Wallis U test. Categorical variables are expressed as numbers (proportions) and were compared using the χ^2^‐test or Fisher's exact test.

First, patients participating in this study were divided into four groups according to sex‐specific quartiles of GGT, which was assessed on admission. Then, the association of GGT levels with stroke recurrence, ischemic stroke, and combined vascular events was estimated by using a multivariate Cox regression model. In addition, hazard ratios (HRs) with 95% confidence intervals (CIs) were calculated after adjusting for potential confounding factors. The first model was adjusted for age and sex. In the second model, body mass index (BMI), smoking status, alcohol consumption and medical histories were added. In the third model, systolic blood pressure (SBP), diastolic blood pressure (DBP), the NIHSS score at admission, the mRS score, the TOAST classification (including five subtypes of ischemic stroke: large‐artery atherosclerosis, cardioembolism, small‐vessel occlusion, other determined etiologies, and undetermined causes),^27^ medications received during hospitalization (including antiplatelet therapy, anticoagulation treatment, antihypertensive treatment, and lipid‐lowering drugs), and laboratory tests were further added. In addition, restricted cubic spline analysis was used to address the association. Furthermore, we used Kaplan–Meier analysis to address survival plots of time to stroke recurrence and other vascular events during the 1‐year follow‐up period, and each group was compared by the log‐rank test. Discrimination tests (C statistics and net reclassification improvement [NRI]) were used to evaluate whether GGT activity improve risk prediction for stroke adverse outcomes. We established the conventional model using various parameters, including age, sex, body mass index, smoking status, NIHSS score at admission, and a medical history of stroke, hypertension, dyslipidemia, diabetes mellitus and cardiovascular diseases.^16^, ^20^ Then, the interaction of age (<60 or ≥60 years), sex, alcohol consumption and TOAST classification was examined by subgroup analysis, adjusted in the third model. In addition, we further performed sensitivity analyses to validate the robustness of the results. First, the Cox proportional hazard regression model, adjusted for the factors in Model 3 plus liver diseases (including liver dysfunction and cirrhosis), was applied. Second, the restricted analysis was performed by excluding individuals with liver diseases (including liver dysfunction and cirrhosis) at baseline. Third, the competing risk model was applied to assess the associations between GGT levels and stroke adverse outcomes, with non‐CVD death being regarded as a competing risk event. In this study, *p*‐values < 0.05 were considered to be statistically significant and were two‐sided.

All statistical analyses were conducted using SAS version 9.4 (SAS Institute Inc.).

## 3 RESULTS

### 3.1 Baseline characteristics

**FIGURE 1:**
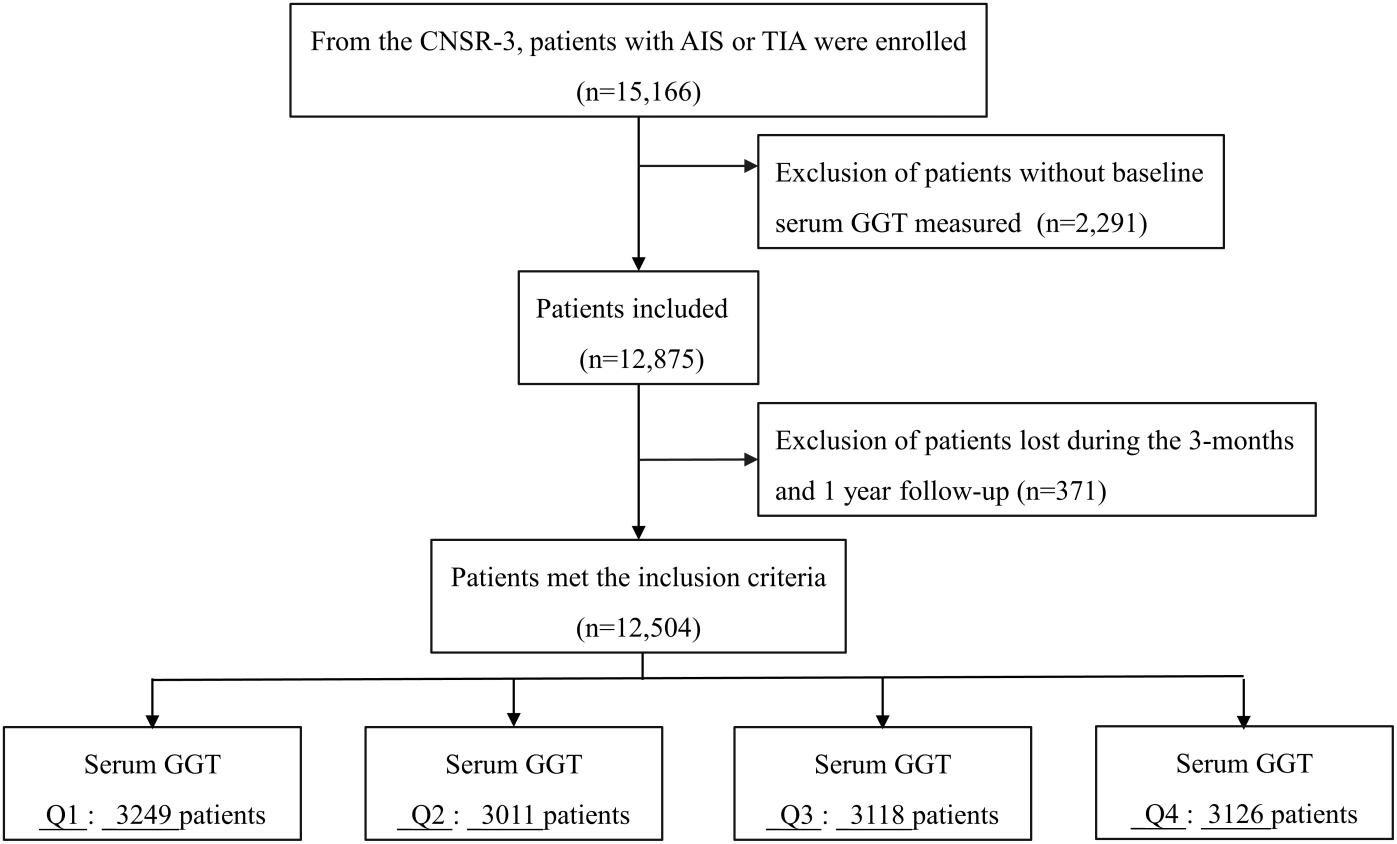
Flowchart in this study. GGT, gamma‐glutamyl transferase

As shown in Figure 1, among the 15,166 patients in the CNSR‐3 study, 12,504 participants who had baseline GGT measurements and completed the 1‐year follow‐up were enrolled, and their baseline characteristics are shown in Table 1 and Table S1. The participants were categorized into four groups according to sex‐specific quartiles of GGT at baseline (men: <19 IU/L, 19–27 IU/L, 27–43 IU/L, ≥43 IU/L; women: <14 IU/L, 14–20 IU/L, 20–29 IU/L, ≥29 IU/L). We found that patients with higher GGT levels were predominantly male and younger overall and were more likely to be obese, be current smokers and drinkers and have higher blood pressure and FBG, TC, TG, ALT, and AST levels.

**TABLE 1:** Baseline characteristics of enrolled patients according to GGT quartile

Characteristic	Total	GGT level				*p* Value
		Q1	Q2	Q3	Q4	
*N*, (%)	12,504	3291	3098	3080	3035	
Age, year, median (IQR)	63.00 (54.00–70.00)	65.00 (58.00–73.00)	64.00 (56.00–71.00)	61.00 (53.00–69.00)	60.00 (52.00–68.00)	<0.0001
Male, *n* (%)	8506 (68.03)	2288 (69.52)	2011 (64.91)	2147 (69.71)	2060 (67.87)	<0.0001
BMI, kg/m^2^, median (IQR)	24.49 (22.60–26.50)	23.88 (22.03–25.80)	24.49 (22.60–26.56)	24.77 (22.99–26.80)	24.80 (22.99–27.03)	<0.0001
BP, mmHg, median (IQR)						
SBP	148.00 (135.00–163.00)	146.50 (134.00–161.50)	148.00 (134.50–163.50)	149.00 (135.00–163.50)	149.00 (135.00–165.00)	0.0033
DBP	86.00 (79.00–95.00)	84.50 (77.50–92.50)	85.00 (77.50–92.50)	85.00 (79.00–94.00)	87.00 (79.5.00–96.00)	<0.0001
Current smoking, *n* (%)	10,399 (83.17)	2715 (82.50)	2600 (83.93)	2533 (82.24)	2551 (84.05)	0.1158
Current drinking, *n* (%)	5559 (44.46)	1308 (39.74)	1272 (41.06)	1449 (47.05)	1530 (50.41)	<0.0001
Medical histories, *n* (%)						
Stroke or TIA	2781 (22.24)	787 (23.91)	731 (23.60)	674 (21.88)	589 (19.41)	<0.0001
Hypertension	7850 (62.78)	1923 (58.43)	1999 (64.53)	1980 (64.29)	1948 (64.18)	<0.0001
Diabetes mellitus	2924 (23.38)	636 (19.33)	792 (25.56)	761 (24.71)	735 (24.22)	<0.0001
Dyslipidemia	980 (7.84)	221 (6.72)	239 (7.71)	265 (8.60)	255 (8.40)	0.0218
Cardiovascular disease	1687 (13.49)	390 (11.85)	396 (12.78)	409 (13.28)	492 (16.21)	<0.0001
Peripheral vascular disease	88 (0.70)	29 (0.88)	24 (0.77)	15 (0.49)	20 (0.66)	0.2766
Liver dysfunction	65 (0.52)	11 (0.33)	8 (0.26)	11 (0.36)	35 (1.15)	<0.0001
Cirrhosis	26 (0.21)	5 (0.15)	7 (0.23)	5 (0.16)	9 (0.30)	0.5718
Medications during hospitalization						
Antiplatelet therapy	12,057 (97.12)	3196 (97.83)	2986 (97.26)	2979 (97.26)	2896 (96.05)	0.0003
Anticoagulation treatment	1294 (10.42)	281 (8.60)	300 (9.77)	356 (11.62)	357 (11.84)	<0.0001
Antihypertensive treatment	5705 (45.95)	1373 (42.03)	1441 (46.94)	1393 (45.48)	1498 (49.68)	<0.0001
Lipid‐lowering drugs	11,942 (96.19)	3144 (96.24)	2954 (96.22)	2956 (96.51)	2888 (95.79)	0.5327
TOAST type, *n* (%)						
Large‐artery atherosclerosis	3170 (25.35)	815 (24.76)	798 (25.76)	814 (26.43)	743 (24.48)	<0.0001
Cardioembolism	769 (6.15)	162 (4.92)	165 (5.33)	188 (6.10)	254 (8.37)	
Small‐vessel occlusion	2589 (20.71)	748 (22.73)	644 (20.79)	609 (19.77)	588 (19.37)	
Other determined etiology	164 (1.31)	61 (1.85)	42 (1.36)	27 (0.88)	34 (1.12)	
Undetermined cause	5812 (46.48)	1505 (45.73)	1449 (46.77)	1442 (46.82)	1416 (46.66)	
NIHSS at admission, median (IQR)	3.00 (1.00–6.00)	3.00 (1.00–6.00)	3.00 (1.00–6.00)	3.00 (1.00–6.00)	3.00 (1.00–6.00)	0.2036
Prestroke mRS≤1, *n* (%)	6290 (50.30)	1623 (49.32)	1585 (51.16)	1573 (51.07)	1509 (49.72)	0.3426
Laboratory tests, median (IQR)						
Serum GGT, IU/L	24.00 (17.00–38.87)	14.00 (12.00–17.00)	21.00 (19.00–24.00)	31.00 (27.00–36.00)	57.00 (46.00–81.00)	<0.0001
FBG, mmol/L	5.51 (4.90–6.85)	5.21 (4.73–6.07)	5.50 (4.88–6.70)	5.62 (4.97–7.10)	5.90 (5.07–7.78)	<0.0001
TC, mmol/L	3.97 (3.31–4.72)	3.77 (3.16–4.50)	3.94 (3.30–4.67)	4.06 (3.38–4.80)	4.16 (3.45–4.98)	<0.0001
TG, mmol/L	1.37 (1.03–1.88)	1.18 (0.91–1.57)	1.33 (1.03–1.77)	1.44 (1.10–2.01)	1.58 (1.18–2.24)	<0.0001
ALT, IU/L	18.00 (13.00–25.00)	14.00 (11.00–18.80)	16.10 (12.00–22.00)	19.10 (14.10–26.00)	25.00 (18.00–37.00)	<0.0001
AST, IU/L	19.00 (16.00–24.00)	17.10 (14.50–21.00)	18.00 (15.00–22.00)	19.00 (16.00–24.00)	23.00 (18.00–30.00)	<0.0001

*Note*: Variables are expressed as median (s) or percentages. GGT was expressed as sex‐specific quartiles (male: Q1, <19 IU/L; Q2, 19–27 IU/L; Q3, 27–43 IU/L; Q4, ≥43 IU/L; female: Q1, <14 IU/L; Q2, 14–20 IU/L; Q3, 20–29 IU/L; Q4, ≥29 IU/L). Cardiovascular disease included coronary heart disease, atrial fibrillation, and heart failure. Medication use indicated treatment during hospitalization.

Abbreviations: ALT, alanine aminotransferase; AST, aspartate aminotransferase; BMI, body mass index; BP, blood pressure; DBP, diastolic blood pressure; FBG, fasting blood glucose; GGT, gamma‐glutamyl transferase; IQR, interquartile range; mRS, the modified Rankin Scale; NIHSS, the National Institutes of Health Stroke Scale; Q, quartile; SBP, systolic blood pressure; TC, total cholesterol; TG, triglycerides; TIA, transient ischemic attack.

### 3.2 Clinical outcomes

**FIGURE 2:**
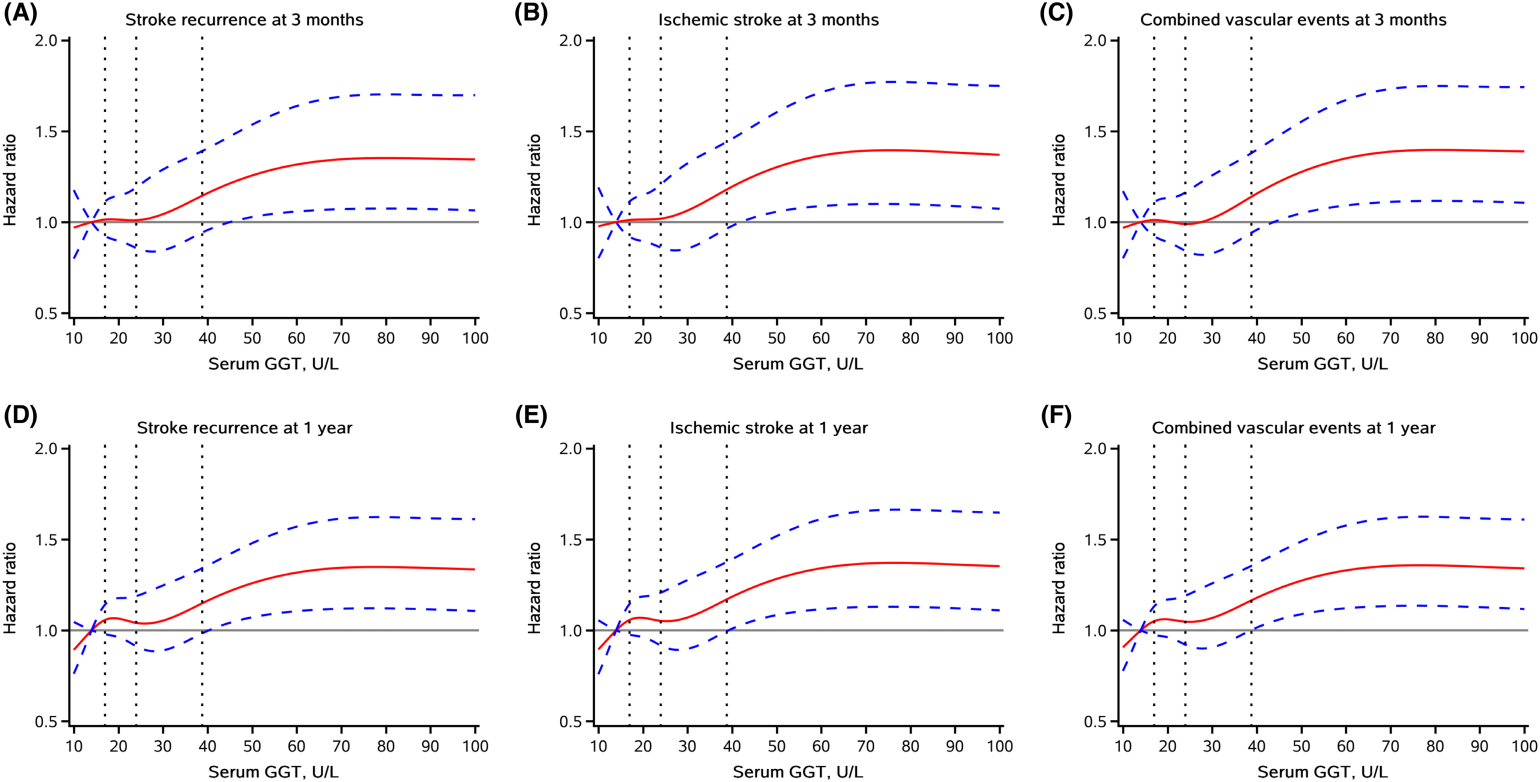
Spline models of the association between GGT levels and stroke adverse outcomes. The association between GGT levels and (A) stroke recurrence, (B) ischemic stroke, and (C) combined vascular events at 3 months is shown in the first line, and the relationship between GGT and (D) stroke recurrence, (E) ischemic stroke, and (F) combined vascular events at the 1‐year follow‐up is shown in the second line. The hazard ratios from the Cox regression model were adjusted for the variables in model 3 in Table 2. The red lines indicate the adjusted hazard ratio, and the blue lines indicate the 95% confidence interval.

During the 1‐year follow‐up period, among the eligible participants, 787 (6.29%) patients had stroke recurrence, 736 (5.89%) had ischemic stroke, and 815 (6.52%) had combined vascular events within 3 months. At the 1‐year follow‐up, 1235 (9.88%), 1134 (9.07%), and 1307 (10.45%) patients had recurrent stroke, ischemic stroke, and combined vascular events, respectively. The associations of GGT levels with stroke adverse outcomes are presented in Table 2. At the 3‐month follow‐up, compared with those in the lowest group, patients in the highest GGT level group showed 32%, 37%, and 34% increases in recurrent stroke [HR (95% CI): 1.32 (1.07–1.63)], ischemic stroke [HR (95% CI): 1.37 (1.10–1.71)], and combined vascular events [HR (95% CI): 1.34 (1.09–1.65)]. The association persisted, even after adjusting for the potential confounding factors mentioned above. Moreover, this positive association remained after the 1‐year follow‐up, and elevated GGT levels revealed adjusted hazard ratios of 1.34 (95% CI: 1.13–1.60), 1.37 (95% CI: 1.14–1.64), and 1.34 (95% CI: 1.13–1.59) for stroke recurrence, ischemic stroke, and combined vascular events, respectively. Notably, the restricted cubic spline analysis also demonstrated an increase in the risk of stroke adverse outcomes with increasing GGT levels (Figure 2).

**TABLE 2:** Associations between GGT levels and stroke adverse outcomes at the 3‐month and 1‐year follow‐ups

Outcomes	GGT quartiles				*p* for trend
	Q1	Q2	Q3	Q4	
3 months					
Stroke recurrence					
Case, *n* (%)	185 (5.62)	192 (6.20)	191 (6.20)	219 (7.22)	
Model 1^a^	1	1.12 (0.92–1.37)	1.15 (0.94–1.41)	1.36 (1.12–1.66)	0.0027
Model 2^b^	1	1.10 (0.89–1.34)	1.12 (0.91–1.37)	1.31 (1.08–1.60)	0.0092
Model 3^c^	1	1.07 (0.88–1.32)	1.09 (0.89–1.34)	1.32 (1.07–1.63)	0.0165
Ischemic stroke					
Case, *n* (%)	171 (5.20)	183 (5.91)	177 (5.75)	205 (6.75)	
Model 1	1	1.15 (0.94–1.42)	1.16 (0.94–1.43)	1.38 (1.12–1.69)	0.0037
Model 2	1	1.13 (0.91–1.39)	1.12 (0.91–1.39)	1.33 (1.08–1.63)	0.0114
Model 3	1	1.11 (0.90–1.37)	1.10 (0.89–1.36)	1.37 (1.10–1.71)	0.0099
Combined vascular events					
Case, *n* (%)	193 (5.86)	196 (6.33)	195 (6.33)	231 (7.61)	
Model 1	1	1.10 (0.90–1.34)	1.14 (0.93–1.39)	1.39 (1.15–1.69)	0.0010
Model 2	1	1.08 (0.88–1.32)	1.11 (0.90–1.35)	1.35 (1.11–1.64)	0.0033
Model 3	1	1.06 (0.86–1.29)	1.07 (0.88–1.32)	1.34 (1.09–1.65)	0.0089
1 year					
Stroke recurrence					
Case, *n* (%)	282 (8.57)	313 (10.10)	305 (9.90)	335 (11.04)	
Model 1	1	1.21 (1.03–1.42)	1.22 (1.04–1.44)	1.39 (1.18–1.63)	0.0001
Model 2	1	1.19 (1.01–1.40)	1.20 (1.02–1.41)	1.36 (1.16–1.60)	0.0004
Model 3	1	1.17 (1.00–1.38)	1.17 (0.99–1.38)	1.34 (1.13–1.60)	0.0020
Ischemic stroke					
Case, *n* (%)	257 (7.81)	289 (9.33)	280 (9.09)	308 (10.15)	
Model 1	1	1.22 (1.03–1.45)	1.23 (1.04–1.46)	1.40 (1.18–1.65)	0.0002
Model 2	1	1.20 (1.02–1.42)	1.20 (1.01–1.43)	1.37 (1.15–1.62)	0.0006
Model 3	1	1.19 (1.00–1.41)	1.18 (0.99–1.40)	1.37 (1.14–1.64)	0.0014
Combined vascular events					
Case, *n* (%)	297 (9.02)	332 (10.72)	325 (10.55)	353 (11.63)	
Model 1	1	1.23 (1.05–1.43)	1.25 (1.06–1.46)	1.40 (1.20–1.64)	<0.0001
Model 2	1	1.21 (1.03–1.41)	1.22 (1.04–1.43)	1.37 (1.17–1.60)	0.0002
Model 3	1	1.18 (1.01–1.39)	1.19 (1.01–1.40)	1.34 (1.13–1.59)	0.0012

*Note*: HRs with 95% CIs were expressed by using multivariate Cox regression models. The HR of quartile 1 was set as the reference. GGT was expressed as sex‐specific quartiles (men: Q1, <19 IU/L; Q2, 19–27 IU/L; Q3, 27–43 IU/L; Q4, ≥43 IU/L; women: Q1, <14 IU/L; Q2, 14–20 IU/L; Q3, 20–29 IU/L; Q4, ≥29 IU/L). Combined vascular events (stroke recurrence, myocardial infarction, and vascular death).

Abbreviations: BMI, body mass index; CI, confidence interval; DBP, diastolic blood pressure; HR, hazard ratio; mRS, the modified Rankin Scale; NIHSS, the National Institutes of Health Stroke Scale; Q, quartile; SBP, systolic blood pressure.

^a^ Model 1 was adjusted for age and sex.

^b^ Model 2 was adjusted for the factors in model 1 plus BMI, smoking, alcohol consumption, and medical histories.

^c^ Model 3 was adjusted for the factors in model 2 plus SBP, DBP, NIHSS score at admission, prestroke mRS score, TOAST types (including large‐artery atherosclerosis, cardioembolism, small‐vessel occlusion, other determined etiology and undetermined cause), medications during hospitalization (including antiplatelet therapy, anticoagulation treatment, antihypertensive treatment, lipid‐lowering drugs), and laboratory tests (fasting blood glucose, total cholesterol, triglycerides, alanine aminotransferase, aspartate aminotransferase levels).

**FIGURE 3:**
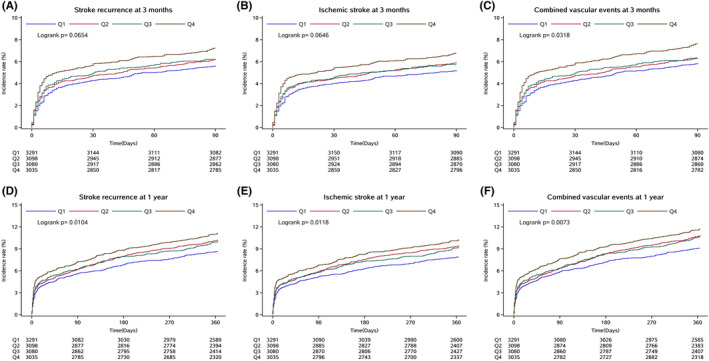
Cumulative incidence of stroke recurrence and adverse outcomes according to GGT levels. Kaplan–Meier curves show the time to event for (A) stroke recurrence, (B) ischemic stroke, and (C) combined vascular events at 3 months and (D) stroke recurrence, (E) ischemic stroke, and (F) combined vascular events at 1 year according to serum GGT levels.

In addition, according to serum GGT levels, the Kaplan–Meier curves showed the time to event for stroke recurrence, ischemic stroke, and combined vascular events at the 3‐month and 1‐year follow‐ups (Figure 3). The incidence rates of stroke recurrence, ischemic stroke, and combined vascular events were quite different at all times in these 4 groups, especially between Q1 and Q4. The top of the fourth quartile showed the highest cumulative incidence of these outcomes, while the bottom of the fourth quartile showed the lowest cumulative incidence.

After applying discrimination tests, there was a slight improvement in predicting the risk of stroke adverse outcomes, namely, recurrence, ischemic stroke, and combined vascular events, when adding GGT activity into the conventional model (including age, sex, body mass index, smoking status, NIHSS score at admission, and a medical history of stroke, hypertension, dyslipidemia, diabetes mellitus, and cardiovascular diseases) [3 months: NRI 14.00% (*p* = 0.0002), NRI 14.00% (*p* = 0.0003), NRI 14.00% (*p* < 0.0001); 1 year: NRI 11.00% (*p* = 0.0005), NRI 11.00% (*p* = 0.0003), NRI 10.00% (*p* = 0.0012), respectively] (Table 3).

**TABLE 3:** Reclassification and disclination statistics for stroke adverse outcomes when adding to GGT levels

Clinical outcomes	Model	C‐statistic		NRI	
		Estimate (95% CI)	*p* Value	Estimate (95% CI)	*p* Value
3 months					
Stroke recurrence	Conventional model	0.64 (0.62–0.66)	0.12	Ref.	0.0002
	Conventional model +GGT	0.65 (0.62–0.67)		0.14 (0.07–0.21)	
Ischemic stroke	Conventional model	0.64 (0.62–0.66)	0.11	Ref.	0.0003
	Conventional model +GGT	0.65 (0.63–0.66)		0.14 (0.07–0.21)	
Combined vascular events	Conventional model	0.64 (0.62–0.66)	0.11	Ref.	<0.0001
	Conventional model +GGT	0.65 (0.63–0.66)		0.14 (0.07–0.21)	
1 year					
Stroke recurrence	Conventional model	0.62 (0.60–0.64)	0.13	Ref.	0.0005
	Conventional model +GGT	0.63 (0.61–0.64)		0.11 (0.05–0.16)	
Ischemic stroke	Conventional model	0.62 (0.60–0.64)	0.11	Ref.	0.0003
	Conventional model +GGT	0.63 (0.61–0.64)		0.11 (0.05–0.17)	
Combined vascular events	Conventional model	0.62 (0.60–0.64)	0.18	Ref.	0.0012
	Conventional model +GGT	0.63 (0.61–0.65)		0.10 (0.04–0.15)	

*Note*: Conventional model: added to factor‐adjusted models, including age, sex, body mass index, smoking, NIHSS score at admission, medical history of stroke, hypertension, dyslipidemia, diabetes mellitus, cardiovascular diseases.

Abbreviations: CI, confidence interval; IDI, integrated discrimination improvement; NRI, net reclassifcation improvement.

According to previous studies,^16^, ^28^, ^29^, ^30^ some demographic and physiological factors might result in different effects of GGT activity on stroke adverse outcomes. Thus, we further conducted an interaction analysis in this study. After stratifying by age, sex, alcohol consumption, and TOAST type, the analysis showed no significant interaction between the variables and GGT activity and the occurrence of stroke adverse outcomes (Table S2). Notably, low‐ and high‐GGT levels refer to the lowest quartile of 25% and the remaining quartiles of 75%, respectively.

In addition, the sensitivity analyses adjusted for the factors in Model 3 plus liver diseases (Table S3), excluding individuals with liver diseases at baseline (Table S4), and the competing risk model (Table S5), all generated similar findings to the primary analysis.

## 4 DISCUSSION

This large cohort study revealed a positive association between baseline serum biomarker GGT levels and stroke adverse outcomes, namely, recurrence, ischemic stroke, and combined vascular events, regardless of the length of time after stroke. This significant relationship persisted even after adjusting for other vascular risk factors, such as age, sex, BMI, alcohol consumption, blood pressure, hypertension, dyslipidemia, diabetes mellitus, cardiovascular disease, TOAST type, medical histories, medications received during hospitalization, and laboratory tests. These trends remained when the models were subjected to multiple sensitivity analyses. Interestingly, adding GGT activity into the conventional model resulted in a 10%–14% increase in predicting stroke adverse outcomes. Moreover, after using correlation analysis to further observe the association between GGT activity and stroke recurrence, ischemic stroke, and combined vascular events in different subgroups, we found that the effect of GGT activity on these adverse outcomes remained consistent.

As a common adverse outcome after stroke, stroke recurrence seriously affects the quality of life of patients, but few studies have examined the role of GGT in stroke recurrence. Several studies indicated that elevated GGT levels were associated with future stroke risk,^16^, ^28^, ^31^, ^32^ but these were all community studies that focused on healthy populations. A study with a small sample size observed that a high‐GGT level was related to all‐cause mortality in stroke patients.^20^ However, the relationship between GGT activity and stroke recurrence was not explored.

The strength of this large, multicenter cohort study is that it demonstrated the association between GGT activity and stroke adverse outcomes, namely, recurrence, ischemic stroke, and combined vascular events, which has rarely been mentioned in previous studies. Due to the significant sex difference in GGT levels, we divided GGT into four levels based on sex, eliminating the interference of this factor in the study. We followed these participants for a long period of time and found that elevated GGT levels were always positively associated with the risk of stroke recurrence.

The results of this study could be explained by the following related mechanisms. GGT is an oxidoreductase located on the outer surface of cell membranes and is mainly found in the liver, although it is also widely found in the brain, heart muscle, and vascular endothelial tissues. According to different molecular weights, GGT fractions have been described as big‐GGT (b‐GGT), medium‐GGT (m‐GGT), small‐GGT (s‐GGT), and free‐GGT (f‐GGT).^33^ GGT participates in the catabolism of extracellular glutathione and increases the availability of intracellular synthetic amino acids, thus maintaining the dynamic balance of intracellular glutathione and cysteine concentrations.^10^ Increased circulating GGT activity is a sign of insufficient antioxidant levels and increased oxidative stress, reflecting an increased inflammatory state in vivo.^7^, ^34^ Lee et al.^35^ also found that after adjusting for confounding factors, GGT levels were positively associated with inflammatory indicators such as the white blood cell count and C‐reactive protein levels.

Moreover, oxidative stress events are related to the pathogenesis of atherosclerosis and a series of cardiovascular‐cerebrovascular diseases (CCDs). During cerebrovascular events, the interruption of blood flow leads to cerebral oxygen deprivation, and the energy failure caused by hypoxia destroys the oxidant‐antioxidant balance, leaving the body in a state of oxidative stress.^36^, ^37^ As an important component of the blood antioxidant defense system, glutathione decreases during the post‐acute phase after stroke.^38^, ^39^ Oxidative stress and the related decrease in glutathione levels induce GGT activity. In the process of glutathione decomposition, GGT produces the strong reducing agent’s cysteine and glycine, which, in turn, produce a large number of superoxide anions while reducing Fe^3+^, thus oxidizing low‐density lipoprotein (LDL), which results in the formation of oxidized LDL. In addition, serum GGT partially adsorbs onto circulating LDL, localizing with oxidized LDL and CD68+ foam cells, and has been found within cerebral, carotid, and coronary atherosclerotic plaques.^40^ Paolicchi confirmed this finding by using immunohistochemistry and found that active GGT was present in coronary and carotid atherosclerotic plaques, which was consistent with the distribution of oxidized LDL.^41^ Moreover, in a study of patients undergoing carotid artery excision, scientists found that b‐GGT levels in carotid artery plaques were not only positively associated with plasma b‐GGT levels but were also closely associated with high total cholesterol levels, intraplaque macrophage infiltration, and plaque vulnerability.^42^ These studies demonstrated that GGT was related to biomarkers of atherosclerotic vulnerable plaques, reflecting the degree of atherosclerosis.^12^, ^42^ Furthermore, GGT activity promoted the progression of atherosclerosis, leading to the occurrence of clinical events and negatively affecting prognosis.^43^, ^44^ A meta‐analysis found that higher GGT activity was related to a greater risk of stroke and coronary heart disease (CHD).^45^ Many population‐based studies have indicated that patients with high‐GGT levels are more likely to have abnormal structural changes and dysfunctions in the heart, which further cause heart failure and atrial fibrillation and even increase cardiovascular‐related mortality.^17^, ^30^, ^46^, ^47^ Moreover, the main causes of stroke are atherosclerosis and cardiogenic embolism, and these findings suggested that serum GGT activity was significantly associated with CCD risk. On this basis, the current study further confirmed the role of GGT in stroke recurrence and other vascular events and enriched the application of GGT activity in disease diagnosis.

This study also had some limitations. First, since only patients with minor stroke (a low NIHSS score) and TIA were included in this study, these findings need validation in other cohorts. Second, for the interesting results of this study to be of greater breadth and relevance, further studies with longer follow‐ups should be considered. Third, because of missing data for some variables of interest, the potential impact of residual confounders, such as the location and size of stroke lesions, partial drug use and drug dosage, on the results were not completely excluded. Furthermore, this study collected only baseline GGT measurements; nevertheless, as a biomarker that reflects metabolism, GGT levels could present a dynamic change characteristic. Thus, we cannot rule out the possibility that this change may influence the role of GGT in stroke recurrence to some extent. The effect of dynamic changes in GGT on stroke recurrence needs to be further verified.

Biomarkers for the diagnosis and prognosis of stroke have always been a focus in this field. Recent research suggests that gut microbiomes related to inflammation may serve as biomarkers and therapeutic targets for stroke.^48^, ^49^ After an injury caused by stroke occurs, DAMPs and cytokines are released from the brain, and the brain‐gut axis involved in maintaining homeostasis is activated, causing intestinal inflammation and changes in immune activity and leading to intestinal barrier damage. Dysfunction of the intestinal immune system allows proinflammatory immune T cells to migrate from the gastrointestinal tract to the brain toward the site of injury, activating proinflammatory cytokines and exacerbating neuronal cell death. In addition, the inflammatory response after stroke is associated with long‐term functional outcomes. Lymphocyte subsets are dynamically changing in stroke patients, which can reflect the local inflammation of the central nervous system. It has been found that T lymphocytes can participate in innate and acquired immune responses and can infiltrate into the brain parenchyma to exert proinflammatory and anti‐inflammatory effects and promote brain injury and functional recovery. Among lymphocyte subsets, the percentage of CD4+ Tregs was a more sensitive biomarker to predict the prognosis of stroke and influence treatment strategies, and it had independent predictive ability for admission, discharge and neurological function at 3 months.^50^ Further studies on the correlation between circulating immunoregulatory lymphocytes and stroke deserve more attention. Moreover, a recent study suggested that enlarged perivascular spaces represent lymphatic drainage dysfunction in the body and are considered a feature of brain disorders, including small vascular disease, cognitive impairment, and epilepsy.^51^ The asymmetric distribution of perivascular spaces may be a new imaging biomarker for the development of brain diseases such as post‐stroke epilepsy, and more research is needed in the future. As a component of the blood–brain barrier tight junction protein, serum occludin is also considered one of the biomarkers to predict the prognosis of stroke. A previous study suggested that the elevation of serum occludin is important to judge the damage to the blood–brain barrier in ischemic stroke.^52^ Elevated serum occludin levels at baseline were associated with a higher risk of hemorrhage transformation regardless of whether patients received reperfusion therapy, and it might be an independent risk factor for early neurological deterioration in patients receiving endovascular thrombectomy. Furthermore, the application value of circulating microRNAs as biomarkers in the diagnosis and prognosis of ischemic and hemorrhagic stroke has also been hotly discussed.^53^ In general, there are abundant studies on the correlation between biomarkers and stroke. Further studies are still needed to select biomarkers that are valuable in predicting the prognosis of stroke.

## 5 CONCLUSION

In conclusion, as a low‐cost, measurable and accurate biomarker, GGT activity was positively associated with the risk of stroke adverse outcomes, namely, recurrence, ischemic stroke, and combined vascular events. This suggests that for stroke patients with high‐GGT levels, more effective medical and lifestyle interventions should be performed as early as possible. More studies are still needed to further investigate the pathophysiological mechanism of GGT activity in the stroke population.

## AUTHOR CONTRIBUTIONS

SQL and AXW designed the study and drafted the manuscript. XT and YTZ analyzed the data. AXW and XM contributed to reviewing the statistical problems. SQL and YMZ interpreted the data. YMZ reviewed the manuscript. All authors approved the final version of the manuscript.

## CONFLICT OF INTEREST

All authors have nothing to disclose.

## Supporting information


TABLE S1–S5

**Appendix S1** Supporting information

## Data Availability

The analysis data are owned by China National Clinical Research Center for Neurological Diseases (http://paper.ncrcnd.ttctrc.com/). Data are not available at present but upon reasonable request and with permission.
