# Supplementary material for: Elevated gamma‐glutamyl transferase levels are associated with stroke recurrence after acute ischemic stroke or transient ischemic attack
Source: CNS Neurosci Ther. 2022 Jul 4;28(10):1637–47. doi: 10.1111/cns.13909 (PMC9437228; doi:10.1111/cns.13909)
Supplement: Supplementary file 1 — TABLE S1–S5 [file CNS-28-1637-s001.docx]

**TABLE S1** Baseline characteristics of participants who were included vs excluded in this study

| Characteristic | Total | Included patients | Excluded patients | P value |
| --- | --- | --- | --- | --- |
| N，(%) | 15166 | 12504 | 2662 |  |
| Age, year, median (IQR) | 63.00(54.00-70.00) | 63.00(54.00-70.00) | 62.00(54.00-70.00) | 0.2030 |
| Male, n (%) | 10364(68.34) | 8506(68.03) | 1858(69.80) | 0.0745 |
| BMI, kg/m2, median (IQR) | 24.49(22.60-26.56) | 24.49(22.60-26.50) | 24.61(22.67-26.71) | 0.1192 |
| BP, mmHg, median (IQR) |  |  |  |  |
| SBP | 148.00(135.00-163.50) | 148.00(135.00-163.00) | 149.00(136.00-164.00) | 0.0344 |
| DBP | 86.00(79.00-95.00) | 86.00(79.00-95.00) | 87.50(80.00-97.50) | <0.0001 |
| Current smoking, n (%) | 12598(83.07) | 10399(83.17) | 2199(82.61) | 0.4855 |
| Current drinking, n (%) | 6797(44.82) | 5559(44.46) | 1238(46.51) | 0.0136 |
| Medical history, n (%) |  |  |  |  |
| Stroke or TIA | 3355(22.12) | 2781(22.24) | 574(21.56) | 0.4440 |
| Hypertension | 9494(62.60) | 7850(62.78) | 1644(61.76) | 0.3225 |
| Diabetes mellitus | 3510(23.14) | 2924(23.38) | 586(22.01) | 0.1278 |
| Dyslipidemia | 1191(7.85) | 980(7.84) | 211(7.93) | 0.8770 |
| Cardiovascular disease | 2041(13.46) | 1687(13.49) | 354(13.30) | 0.7906 |
| Peripheral vascular disease | 118(0.78) | 88(0.70) | 30(1.13) | 0.0240 |
| Medications during hospitalization |  |  |  |  |
| Antipaltelet therapy | 14613(97.08) | 12057(97.12) | 2556(96.89) | 0.5336 |
| Anticoagulation treatment | 1546(10.27) | 1294(10.42) | 252(9.55) | 0.1812 |
| Antihypertensive treatment | 7000(46.50) | 5705(45.95) | 1295(49.09) | 0.0033 |
| TOAST types, n (%) |  |  |  | 0.0454 |
| Large-artery atherosclerosis | 3856(25.43) | 3170(25.35) | 686(25.77) |  |
| Cardioembolism | 917(6.05) | 769(6.15) | 148(5.56) |  |
| Small-vessel occlusion | 3165(20.87) | 2589(20.71) | 576(21.64) |  |
| Other determined etiology | 182(1.20) | 164(1.31) | 18(0.68) |  |
| Undetermined cause | 7046(46.46) | 5812(46.48) | 1234(46.36) |  |
| NIHSS at admission, median (IQR) | 3.00(1.00-6.00) | 3.00(1.00-6.00) | 3.00(2.00-6.00) | <0.0001 |
| Prestroke mRS≤1, n (%) | 7501(49.46) | 6290(50.30) | 1211(45.49) | <0.0001 |
| Laboratory tests, median (IQR) |  |  |  |  |
| Serum GGT, IU/L | 24.00(17.00-38.75) | 24.00(17.00-38.87) | 25.00(17.00-38.00) | 0.7499 |
| FBG, mmol/L | 5.52(4.90-6.89) | 5.51(4.90-6.85) | 5.58(4.900-7.04) | 0.0995 |
| TC, mmol/L | 3.97(3.31-4.72) | 3.95(3.35-4.70) | 3.97(3.31-4.72) | 0.9172 |
| TG, mmol/L | 1.37(1.03-1.87) | 1.35(1.03-1.84) | 1.37(1.03-1.88) | 0.5346 |
| ALT, IU/L | 18.00(13.00-26.00) | 18.30(13.70-27.40) | 18.00(13.00-25.00) | 0.0003 |
| AST, IU/L | 19.00(16.00-24.00) | 20.00(16.00-24.80) | 19.00(16.00-24.00) | <0.0001 |

Variables are expressed as median(s) or percentages. Cardiovascular disease included atrial fibrillation, coronary heart disease, and heart failure. Medication use indicated treatment during hospitalization. BMI, body mass index; BP, blood pressure; SBP, systolic blood pressure; DBP, diastolic blood pressure; TIA, transient ischemic attack; NIHSS, the National Institutes of Health Stroke Scale; mRS, the modified Rankin Scale; GGT, gamma-glutamyl transferase; FBG, fasting blood glucose; TC, total cholesterol; TG, triglycerides; ALT, alanine aminotransferase; AST, aspartate aminotransferase; IQR, interquartile range.

**TABLE S2** Subgroup analysis of the association between GGT and stroke outcomes at the follow-up

|  | Stroke recurrence | | | | Ischemic stroke | | | | Combined vascular events | | | |
| --- | --- | --- | --- | --- | --- | --- | --- | --- | --- | --- | --- | --- |
|  | Low-GGT  No. (%) | High-GGT  No. (%) | HR (95%) | P_interraction_ | Low-GGT  No. (%) | High-GGT  No. (%) | HR (95%) | P_interraction_ | Low-GGT  No. (%) | High-GGT  No. (%) | HR (95%) | P_interraction_ |
| 3-months |  |  |  |  |  |  |  |  |  |  |  |  |
| Age, years |  |  |  |  |  |  |  |  |  |  |  |  |
| <60 | 55 (5.55) | 228(5.91) | 1.02(0.75-1.40) | 0.66 | 52(5.25) | 213(5.53) | 1.02(0.74-1.40) | 0.50 | 55(5.55) | 232(6.02) | 1.03(0.76-1.40) | 0.77 |
| ≥60 | 130(5.65) | 374(6.98) | 1.19(0.96-1.46) |  | 119(5.17) | 352(6.57) | 1.22(0.98-1.52) |  | 138(6.00) | 390(7.28) | 1.17(0.95-1.43) |  |
| Sex |  |  |  |  |  |  |  |  |  |  |  |  |
| male | 128(5.59) | 384(6.18) | 1.03(0.84-1.28) | 0.53 | 116(5.07) | 354(5.69) | 1.07(0.86-1.33) | 0.56 | 135(5.90) | 397(6.38) | 1.02(0.83-1.26) | 0.44 |
| female | 57(5.68) | 218(7.28) | 1.33(0.98-1.80) |  | 55(5.48) | 211(7.05) | 1.35(0.99-1.83) |  | 58(5.78) | 225(7.51) | 1.35(1.00-1.82) |  |
| Alcohol consumption |  |  |  |  |  |  |  |  |  |  |  |  |
| None | 30(5.26) | 157(6.68) | 1.15(0.77-1.74) | 0.51 | 27(4.74) | 146(6.21) | 1.26(0.82-1.93) | 0.46 | 32(5.61) | 161(6.85) | 1.12(0.75-1.66) | 0.59 |
| Yes | 155(5.70) | 445(6.48) | 1.15(0.95-1.40) |  | 144(5.29) | 419(6.11) | 1.17(0.96-1.42) |  | 161(5.92) | 461(6.72) | 1.15(0.96-1.39) |  |
| TOAST types |  |  |  |  |  |  |  |  |  |  |  |  |
| Large-artery atherosclerosis | 65(7.98) | 215(9.13) | 1.20(0.90-1.61) | 0.79 | 59(7.24) | 206(8.75) | 1.28(0.95-1.74) | 0.85 | 67(8.22) | 222(9.43) | 1.19(0.90-1.59) | 0.74 |
| Cardioembolism | 9(5.56) | 43(7.08) | 1.60(0.74-3.44) |  | 8(4.94) | 36(5.93) | 1.55(0.69-3.50) |  | 10(6.17) | 47(7.74) | 1.52(0.74-3.14) |  |
| Small-vessel occlusion | 25(3.34) | 88(4.78) | 1.31(0.82-2.08) |  | 23(3.07) | 80(4.35) | 1.28(0.79-2.10) |  | 26(3.48) | 91(4.94) | 1.31(0.83-2.07) |  |
| Other determined etiology | 4(6.56) | 9(8.74) | 2.65(0.48- 14.61) |  | 4(6.56) | 9(8.74) | 2.65(0.48-14.61) |  | 4(6.56) | 9(8.74) | 2.65(0.48-14.61) |  |
| Undetermined cause | 82(5.45) | 247(5.73) | 1.03(0.79-1.34) |  | 77(5.12) | 234(5.43) | 1.04(0.80-1.35) |  | 86(5.71) | 253(5.87) | 1.01(0.78-1.31) |  |
|  |  |  |  |  |  |  |  |  |  |  |  |  |
|  |  |  |  |  |  |  |  |  |  |  |  |  |
| 1-year |  |  |  |  |  |  |  |  |  |  |  |  |
| Age, years |  |  |  |  |  |  |  |  |  |  |  |  |
| <60 | 85(8.58) | 345(8.95) | 1.03(0.80-1.32) | 0.19 | 78(7.87) | 316(8.20) | 1.04(0.80-1.35) | 0.18 | 88(8.88) | 359(9.31) | 1.03(0.81-1.32) | 0.17 |
| ≥60 | 197(8.57) | 608(11.35) | 1.28(1.08-1.52) |  | 179(7.78) | 561(10.47) | 1.30(1.09-1.55) |  | 209(9.09) | 651(12.15) | 1.30(1.11-1.54) |  |
| Sex |  |  |  |  |  |  |  |  |  |  |  |  |
| male | 204(8.92) | 615(9.89) | 1.09(0.93-1.29) | 0.12 | 183(8.00) | 556(8.94) | 1.11(0.93-1.32) | 0.14 | 212(9.27) | 658(10.58) | 1.14(0.97-1.34) | 0.32 |
| female | 78(7.78) | 338(11.29) | 1.46(1.13-1.89) |  | 74(7.38) | 321(10.72) | 1.47(1.13-1.92) |  | 85(8.47) | 352(11.75) | 1.40(1.10- 1.80) |  |
| Alcohol consumption |  |  |  |  |  |  |  |  |  |  |  |  |
| None | 48(8.42) | 245(10.42) | 1.20(0.87-1.66) | 0.64 | 43(7.54) | 224(9.53) | 1.28(0.91-1.80) | 0.58 | 50(8.77) | 259(11.02) | 1.23(0.90-1.69) | 0.57 |
| Yes | 234(8.60) | 708(10.32) | 1.22(1.04- 1.42) |  | 214(7.86) | 653(9.52) | 1.22(1.04-1.44) |  | 247(9.08) | 751(10.94) | 1.23(1.06-1.43) |  |
| TOAST types |  |  |  |  |  |  |  |  |  |  |  |  |
| Large-artery atherosclerosis | 89(10.92) | 323(13.72) | 1.34(1.05- 1.71) | 0.58 | 82(10.06) | 304(12.91) | 1.37(1.06-1.77) | 0.57 | 93(11.41) | 338(14.35) | 1.33(1.05-1.69) | 0.68 |
| Cardioembolism | 17(10.49) | 73(12.03) | 1.25(0.71-2.19) |  | 16(9.88) | 63(10.38) | 1.11(0.62-2.00) |  | 19(11.73) | 83(13.67) | 1.28(0.76-2.18) |  |
| Small-vessel occlusion | 45(6.02) | 148(8.04) | 1.32(0.93- 1.87) |  | 39(5.21) | 129(7.01) | 1.32(0.90-1.92) |  | 47(6.28) | 153(8.31) | 1.30(0.92-1.84) |  |
| Other determined etiology | 8(13.11) | 10(9.71) | 0.86(0.26-2.88) |  | 8(13.11) | 10(9.71) | 0.86(0.26-2.88) |  | 8(13.11) | 11(10.68) | 1.00(0.30-3.32) |  |
| Undetermined cause | 123(8.17) | 399(9.26) | 1.14(0.92-1.41) |  | 112(7.44) | 371(8.61) | 1.17(0.94-1.46) |  | 130(8.64) | 425(9.87) | 1.16(0.94-1.42) |  |

The HRs were adjusted for the variables in model 3 in Table 2. Hazard ratios for GGT and stroke outcomes are stratified by age, sex, alcohol consumption, and TOAST type. Low GGT refers to the lowest quartile of 25%, and high GGT refers to the remaining 75% quartiles. GGT, gamma-glutamyl transferase; BMI, body mass index; SBP, systolic blood pressure; DBP, diastolic blood pressure; HR, hazard ratio; CI, confidence interval.

**TABLE S3** Sensitivity analysis of the association between GGT and stroke outcomes adjusted for the confounding factors including liver diseases

| Outcomes | GGT quartiles | | | | P for trend |
| --- | --- | --- | --- | --- | --- |
|  | Q1 | Q2 | Q3 | Q4 |  |
| 3 months |  |  |  |  |  |
| Stroke recurrence |  |  |  |  |  |
| Model 4 | 1 | 1.07(0.88-1.31) | 1.09(0.88-1.34) | 1.31(1.06-1.63) | 0.0171 |
| Ischemic stroke |  |  |  |  |  |
| Model 4 | 1 | 1.11(0.90-1.37) | 1.10(0.89-1.36) | 1.37(1.10-1.71) | 0.0103 |
| Combined vascular events |  |  |  |  |  |
| Model 4 | 1 | 1.06 (0.86-1.29) | 1.07(0.87-1.32) | 1.34(1.09-1.65) | 0.0092 |
| 1 year |  |  |  |  |  |
| Stroke recurrence |  |  |  |  |  |
| Model 4 | 1 | 1.17(0.99-1.38) | 1.17(0.99-1.38) | 1.34(1.13-1.60) | 0.0020 |
| Ischemic stroke |  |  |  |  |  |
| Model 4 | 1 | 1.18(1.00-1.40) | 1.18(0.99-1.40) | 1.37(1.14-1.64) | 0.0015 |
| Combined vascular events |  |  |  |  |  |
| Model 4 | 1 | 1.18(1.01-1.39) | 1.19(1.01-1.40) | 1.34(1.13-1.59) | 0.0012 |

HRs with 95% CIs were expressed by using multivariate Cox regression models. The HR of quartile 1 was set as the reference. GGT was expressed as sex-specific quartiles (men: Q1, <19 IU/L; Q2, 19-27 IU/L; Q3, 27–43 IU/L; Q4, ≥43 IU/L; women: Q1, <14 IU/L; Q2, 14-20 IU/L; Q3, 20–29 IU/L; Q4, ≥29 IU/L). Combined vascular events (stroke recurrence, myocardial infarction, and vascular death).

Model 4 was adjusted for the factors in model 3 in table 2 plus liver diseases (including liver dysfunction and cirrhosis).

**TABLE S4** Sensitivity analyses for the association of GGT levels with clinical outcomes after exclusion of participants with liver diseases at baseline

| Outcomes | GGT quartiles | | | | P for trend |
| --- | --- | --- | --- | --- | --- |
|  | Q1 | Q2 | Q3 | Q4 |  |
| 3 months |  |  |  |  |  |
| Stroke recurrence |  |  |  |  |  |
| Model 3 | 1 | 1.06(0.87-1.30) | 1.10(0.89-1.35) | 1.32(1.07-1.64) | 0.0140 |
| Ischemic stroke |  |  |  |  |  |
| Model 3 | 1 | 1.10(0.89-1.36) | 1.11(0.89-1.38) | 1.38(1.10-1.72) | 0.0081 |
| Combined vascular events |  |  |  |  |  |
| Model 3 | 1 | 1.05 (0.86-1.28) | 1.08(0.88-1.33) | 1.34(1.09-1.66) | 0.0074 |
| 1 year |  |  |  |  |  |
| Stroke recurrence |  |  |  |  |  |
| Model 3 | 1 | 1.16(0.99-1.37) | 1.16(0.98-1.37) | 1.34(1.13-1.60) | 0.0019 |
| Ischemic stroke |  |  |  |  |  |
| Model 3 | 1 | 1.18(1.00-1.40) | 1.17(0.99-1.40) | 1.37(1.14-1.65) | 0.0014 |
| Combined vascular events |  |  |  |  |  |
| Model 3 | 1 | 1.18(1.00-1.38) | 1.19(1.01-1.39) | 1.34(1.14-1.59) | 0.0011 |

HRs with 95% CIs were expressed by using multivariate Cox regression models. The HR of quartile 1 was set as the reference. GGT was expressed as sex-specific quartiles (men: Q1, <19 IU/L; Q2, 19-27 IU/L; Q3, 27–43 IU/L; Q4, ≥43 IU/L; women: Q1, <14 IU/L; Q2, 14-20 IU/L; Q3, 20–29 IU/L; Q4, ≥29 IU/L). Combined vascular events (stroke recurrence, myocardial infarction, and vascular death).

**TABLE S5** Sensitivity analyses for the association of GGT levels with clinical outcomes after taking non-CVD related death as competing risk event

| Outcomes | GGT quartiles | | | | P for trend |
| --- | --- | --- | --- | --- | --- |
|  | Q1 | Q2 | Q3 | Q4 |  |
| 3 months |  |  |  |  |  |
| Stroke recurrence |  |  |  |  |  |
| Model 3 | 1 | 1.07(0.88-1.31) | 1.09(0.88-1.34) | 1.31(1.05-1.64) | 0.0241 |
| Ischemic stroke |  |  |  |  |  |
| Model 3 | 1 | 1.11(0.90-1.37) | 1.10(0.88-1.36) | 1.37(1.09-1.72) | 0.0150 |
| Combined vascular events |  |  |  |  |  |
| Model 3 | 1 | 1.06 (0.87-1.29) | 1.07(0.87-1.32) | 1.34(1.08-1.67) | 0.0135 |
| 1 year |  |  |  |  |  |
| Stroke recurrence |  |  |  |  |  |
| Model 3 | 1 | 1.17(1.00-1.37) | 1.16(0.98-1.37) | 1.33(1.12-1.59) | 0.0028 |
| Ischemic stroke |  |  |  |  |  |
| Model 3 | 1 | 1.18(1.00-1.40) | 1.17(0.99-1.40) | 1.37(1.14-1.65) | 0.0020 |
| Combined vascular events |  |  |  |  |  |
| Model 3 | 1 | 1.18(1.01-1.38) | 1.18(1.01-1.39) | 1.34(1.13-1.59) | 0.0016 |

HRs with 95% CIs were expressed by using multivariate Cox regression models. The HR of quartile 1 was set as the reference. GGT was expressed as sex-specific quartiles (men: Q1, <19 IU/L; Q2, 19-27 IU/L; Q3, 27–43 IU/L; Q4, ≥43 IU/L; women: Q1, <14 IU/L; Q2, 14-20 IU/L; Q3, 20–29 IU/L; Q4, ≥29 IU/L). Combined vascular events (stroke recurrence, myocardial infarction, and vascular death).
