# Supplementary material for: Elevated gamma‐glutamyl transferase levels are associated with stroke recurrence after acute ischemic stroke or transient ischemic attack
Source: CNS Neurosci Ther. 2022 Jul 4;28(10):1637–47. doi: 10.1111/cns.13909 (PMC9437228; doi:10.1111/cns.13909)

Full unedited gel/blot for Figure 2A

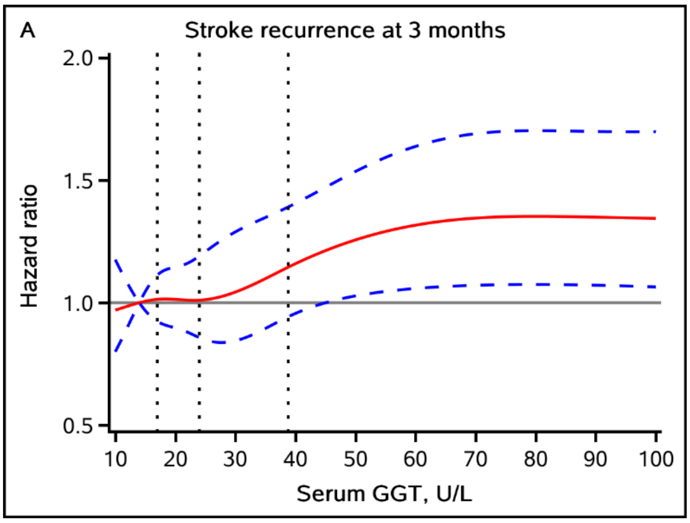

Full unedited gel/blot for Figure 2B

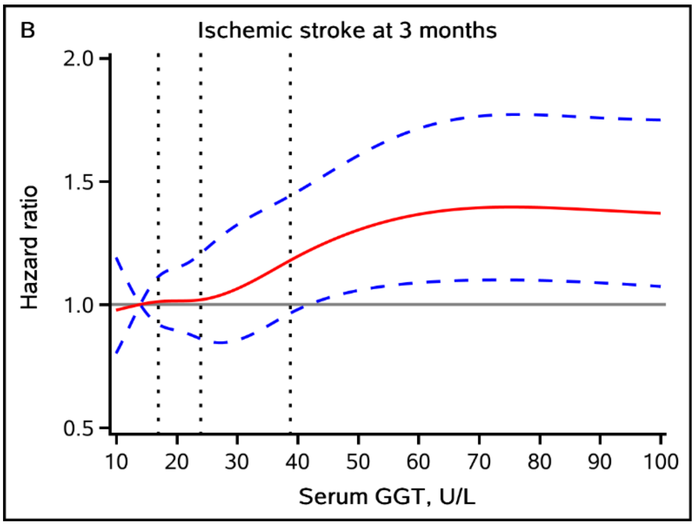

Full unedited gel/blot for Figure 2C

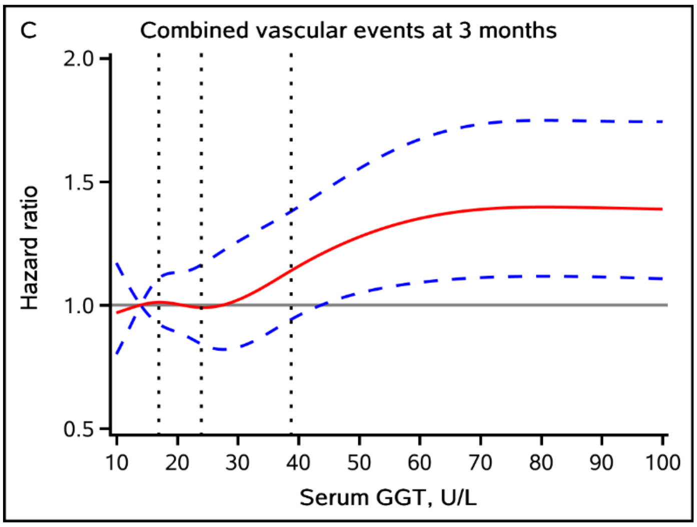

Full unedited gel/blot for Figure 2D

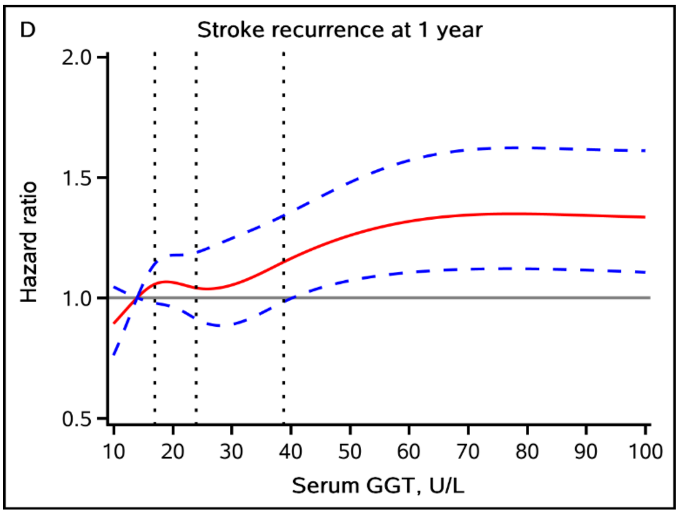

Full unedited gel/blot for Figure 2E

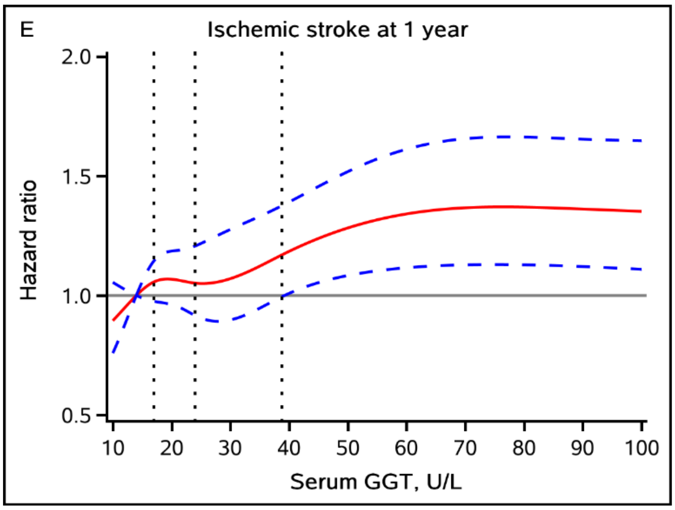

Full unedited gel/blot for Figure 2F

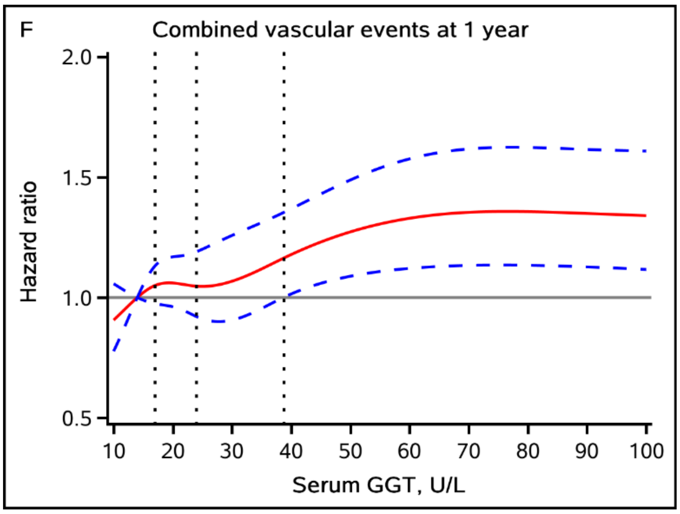

Full unedited gel/blot for Figure 3A

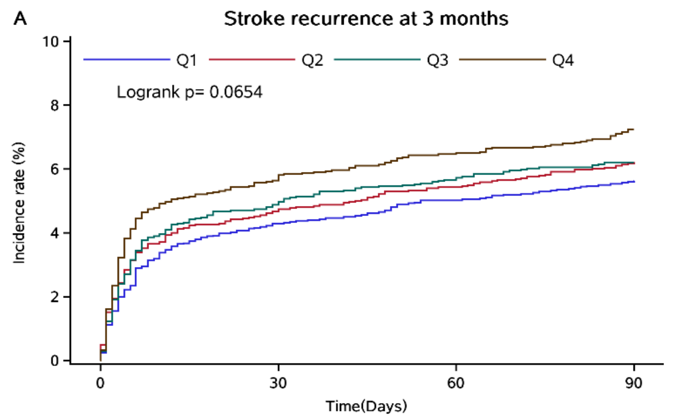

	0	30	60	90
Q1	3291	3144	3111	3082
Q2	3098	2945	2912	2877
Q3	3080	2917	2886	2862
Q4	3035	2850	2817	2785

Full unedited gel/blot for Figure 3B

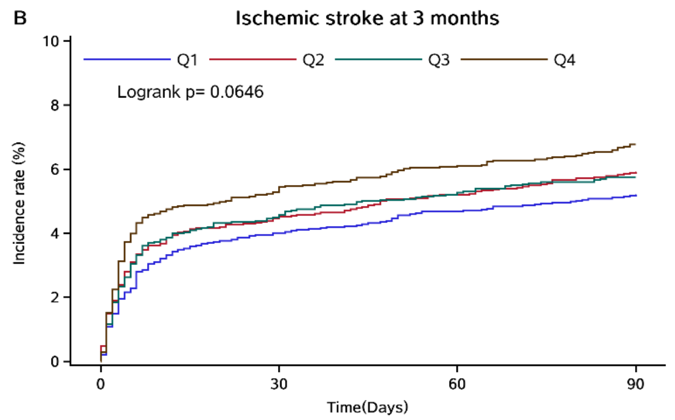

	0	30	60	90
Q1	3291	3150	3117	3090
Q2	3098	2951	2918	2885
Q3	3080	2924	2894	2870
Q4	3035	2859	2827	2796

Full unedited gel/blot for Figure 3C

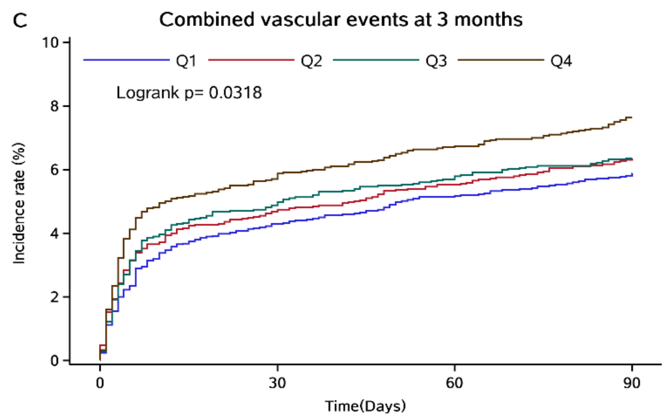

	0	30	60	90
Q1	3291	3144	3110	3080
Q2	3098	2945	2910	2874
Q3	3080	2917	2886	2860
Q4	3035	2850	2816	2782

Full unedited gel/blot for Figure 3D

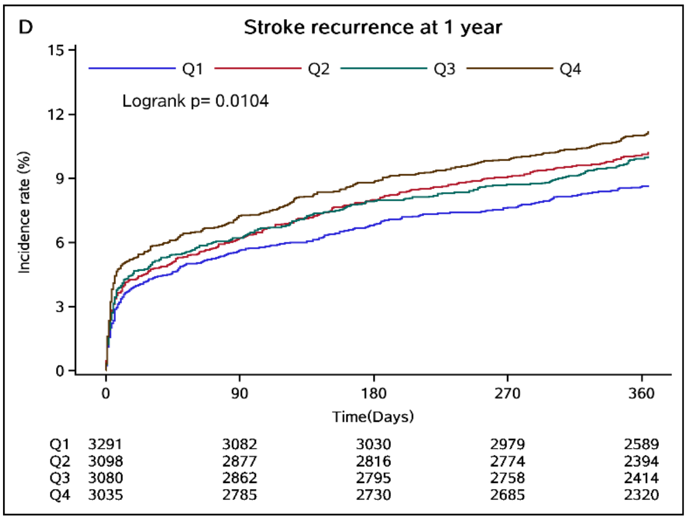

Full unedited gel/blot for Figure 3E

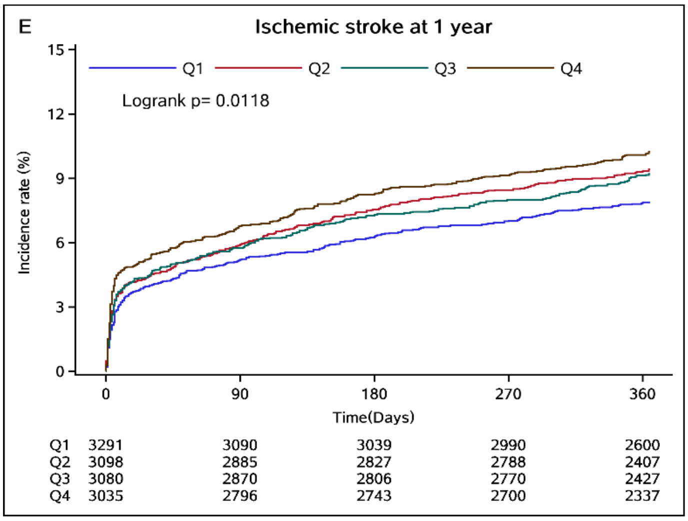

Full unedited gel/blot for Figure 3F

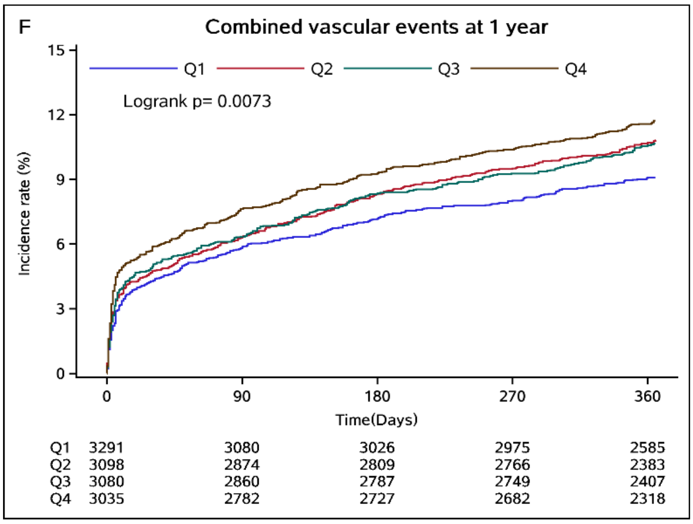

Supplement: Supplementary file 2 — Appendix S1 Supporting information [file CNS-28-1637-s002.pdf]
